# Correction: Al Namat et al. Omphalocele and Associated Anomalies: Exploring Pulmonary Development and Genetic Correlations—A Literature Review. *Diagnostics* 2025, *15*, 675

**DOI:** 10.3390/diagnostics15243210

**Published:** 2025-12-16

**Authors:** Dina Al Namat, Romulus Adrian Roșca, Razan Al Namat, Elena Hanganu, Andrei Ivan, Delia Hînganu, Ancuța Lupu, Marius Valeriu Hînganu

**Affiliations:** 1 Faculty of Medicine, University of Medicine and Pharmacy “Grigore T. Popa”, 700115 Iasi, Romania; dina.rosca-al.namat@umfiasi.ro (D.A.N.); adrianrosca82@yahoo.com (R.A.R.); dr.elenahanganu@gmail.com (E.H.); ivan.andrei89@gmail.com (A.I.); hinganu.delia@umfiasi.ro (D.H.); marius.hinganu@umfiasi.ro (M.V.H.); 2 Department of Surgery II-Pediatric Surgery, 700309 Iasi, Romania; 3 “Saint Mary” Emergency Children Hospital, 700309 Iasi, Romania; 4 Department of Mother and Child Medicine, University of Medicine and Pharmacy “Grigore T. Popa”, 700115 Iasi, Romania; anca_ign@yahoo.com

In the original publication [1], the author Andrei Ivan’s contributions are incomplete. The updated author contributions are as follows: methodology, A.I. and D.A.N.

The mention of “funding acquisition” in the Author Contributions section was an error. This study did not receive any external funding. We removed “funding acquisition, R.A.R. and R.A.N.”.

Two of the cited works (References [124,128]) are irrelevant and off-topic and should be removed. With this correction, the order of some references has been adjusted accordingly.

The authors state that the scientific conclusions are unaffected. This correction was approved by the Academic Editor. The original publication has also been updated.
